# Machine learning approaches to evaluate infants’ general movements in the writhing stage—a pilot study

**DOI:** 10.1038/s41598-024-54297-1

**Published:** 2024-02-24

**Authors:** Lisa Letzkus, J. Vince Pulido, Abiodun Adeyemo, Stephen Baek, Santina Zanelli

**Affiliations:** 1 Department of Pediatrics, University of Virginia Children’s Hospital, PO Box 800828, Charlottesville, VA 22908 USA; 2 Applied Physics Laboratory, Johns Hopkins University, Laurel, MD USA; 3 School of Data Science, University of Virginia, Charlottesville, VA USA

**Keywords:** Predictive markers, Neonatal brain damage

## Abstract

The goals of this study are to describe machine learning techniques employing computer-vision movement algorithms to automatically evaluate infants’ general movements (GMs) in the writhing stage. This is a retrospective study of infants admitted 07/2019 to 11/2021 to a level IV neonatal intensive care unit (NICU). Infant GMs, classified by certified expert, were analyzed in two-steps (1) determination of anatomic key point location using a NICU-trained pose estimation model [accuracy determined using object key point similarity (OKS)]; (2) development of a preliminary movement model to distinguish normal versus cramped-synchronized (CS) GMs using cosine similarity and autocorrelation of major joints. GMs were analyzed using 85 videos from 74 infants; gestational age at birth 28.9 ± 4.1 weeks and postmenstrual age (PMA) at time of video 35.9 ± 4.6 weeks The NICU-trained pose estimation model was more accurate (0.91 ± 0.008 OKS) than a generic model (0.83 ± 0.032 OKS, p < 0.001). Autocorrelation values in the lower limbs were significantly different between normal (5 videos) and CS GMs (5 videos, p < 0.05). These data indicate that automated pose estimation of anatomical key points is feasible in NICU patients and that a NICU-trained model can distinguish between normal and CS GMs. These preliminary data indicate that machine learning techniques may represent a promising tool for earlier CP risk assessment in the writhing stage and prior to hospital discharge.

## Introduction

Cerebral palsy (CP) is the leading childhood motor neuro-disability, affecting 1 in 325 infants in the United States^1^. Advances in neonatal care have led to increased survival of preterm infants, even those born extremely preterm. However, the risk of neurodevelopmental disability and CP, in particular, remains high^2^ with an increased incidence and severity of CP in infants born at younger gestational ages (GA)^3,4^. Research shows that early identification of CP risk followed by early intervention at favorable stages of brain development^5–7^ are critical steps in improving outcomes^6,8^.

Infant general movements (GMs) are a reliable predictor of later motor development and have been used with high accuracy for the early identification of CP risk in high-risk infants^9–11^. Infants typically exhibit these spontaneous GMs through 20 weeks post-term and progress from writhing GMs (9 weeks gestation to 8 weeks post-term) to fidgety GMs (6 to 20 weeks post-term) before transitioning to goal oriented movements^10^. Infants with abnormal GMs are known to be at higher risk of neuro-disability and CP in particular. The Prechtl General Movement Assessment (GMA) is a validated qualitative diagnostic tool developed for the classification of GMs by certified providers. The GMA is non-invasive and can be used shortly after birth to monitor GMs longitudinally with the goal to detect high-risk GM patterns^9–12^. The presence of cramped-synchronized (CS) GMs in the writhing stage followed by absent fidgety GMs in the fidgety stage has a specificity of 96% for the identification of high-risk CP^7,13,14^. Persistent CS GMs are also predictive of later diagnosis of CP with a specificity of 92.5%^12^. The GMA is typically used in combination with other assessments such as brain imaging and physical evaluation in order to make a diagnosis of CP^6^.

While the GMA is an effective tool for the early detection of CP^6^, there are barriers to its widespread implementation into routine neonatal intensive care unit (NICU) clinical care, including associated cost of training and re-training, as well as resource allocation considerations to obtain and score videos^15^. As such, technologies aiming to decrease the impact of these barriers may lead to more neonates benefiting from the GMA. Many investigators have studied sensor-based methods to augment the GMA^16–22^, including accelerometers attached to the infants’ limbs^17,23,24^. However, these sensor-based methods have limited applicability due to concerns regarding skin integrity in small premature infants, aside from the added logistic constraints of setting up sensors for each measurement session^25,26^.

Computer vision and machine learning techniques are increasingly integrated into clinical care. Especially, recent advances in deep convolutional neural networks (CNNs) have enabled accurate, robust, and reliable optical human movement monitoring that do not require any sensors or fiducial markers. For instance, there is emerging evidence that machine learning models can assist with the identification of CP risk, specifically automated pose estimation methods aiming to analyze infants’ movements within the context of the GMA^16,17,19–22,27,28^. With the emphasis on early diagnosis, the development of quantitative and automated methods to accurately identify concerning GMs (specifically CS GMs) early (in the writhing stage) and prior to NICU discharge is a key research priority. These techniques may also bring to light additional and more subtle patterns that could be incorporated into prediction algorithms for other neuro-disabilities^29^. Importantly, the successful development of these techniques may allow for widespread screening of all infants, as the majority of infants are not cared for at centers with expertise in the GMA. However, in the clinical context, further research is needed to bridge currently available movement models to clinical application in the NICU.

In this retrospective cohort pilot study, we describe the use of a machine learning model to analyze infants’ GMs in the writhing stage and during NICU hospitalization in a strictly automated fashion. To achieve this goal, we first trained a pose estimation model on NICU-based images and evaluated its performance against standard out-of-the-box models. Second, using the output of the NICU-trained pose estimation model, we developed a preliminary movement model prototype to differentiate between normal (low-risk) GMs and CS (high-risk) GMs. Results from this study describe the utility of machine learning techniques to accurately differentiate normal from CS GMs using autocorrelation as a measure of repetitive movement. In addition, this study also widens the range of machine learning approaches for the automated analysis of infants’ movement to determine CP risk into earlier age groups.

## Methods

### Sample selection and experimental setting

This clinical observational retrospective pilot study using archival GMA videos was conducted in the level IV NICU at the University of Virginia (UVA). This study was approved by the University of Virginia Human Sciences Research Internal Review Board (UVA-HSR) with waiver of consent and was performed in accordance with relevant guidelines and regulations. At UVA, the GMA is obtained as standard-of-care for the assessment of the motor repertoire of very low birth weight (VLBW) infants [GA < 32 weeks and/or birth weight (BW) < 1500 g] and all infants at high-risk for neuro-disability including those with hypoxic ischemic encephalopathy^5^. The timing of obtaining the GMA videos was not dictated by this study and was obtained per unit guidelines. All GMA videos were obtained in a standardized fashion with infants in supine position and dressed in lightweight clothing with arms and legs bare and with removal of all positioning aids and/or other barriers, to allow for free movement of all extremities. Videos were obtained from a consistent angle, from above the infant and in a vertical orientation. Videos were stopped when at least 3 GMs were observed (5370.93 ± 1353.28 frames or 179.03 ± 45.11 s). Videos were subsequently reviewed and scored on a weekly basis by at least 2 GMA-certified evaluators per unit routine procedure. GMA results were documented in the patients’ electronic medical record and concerning patterns (CS GMs) were directly reported to the treatment team.

Videos used for this study were selected from the GMA video archives housed on a secure UVA network which strictly follows the UVA protocol for electronic storage of highly sensitive data. Infants with archival GMA videos and admitted to the NICU between July 2019 and November 2021 were selected for analysis if they met the quality checks discussed above.

### Annotation process

Current open-source, state-of-the-art pose estimation algorithms are not trained for infant body shapes and/or for complex imaging conditions such as those occurring in the NICU setting, raising concerns of sub-optimal performance. Hence, we first constructed a NICU-based training dataset to fine-tune an already available pose estimation model, the Microsoft Common Objects in Context (MS COCO) dataset^30^. Using the Visipedia Annotation Toolkit as the primary labeling software (https://github.com/visipedia/annotation_tools), a team of annotators (LL, SZ, VP, AM, SP) manually labeled 17 anatomical key points: eyes, nose, ears, shoulders, elbows, wrists, hips, knees, and ankles of the infants. All videos were labeled by a single human annotator. For annotation, we sampled every 10 other frames from each of the videos that had 30 frames per second (fps). For example, if a video contains 100 frames, we sample every 10th frame for annotation. This sampling method yielded 620 images for training and 140 images for testing.

### Training and test dataset development

**Figure 1:**
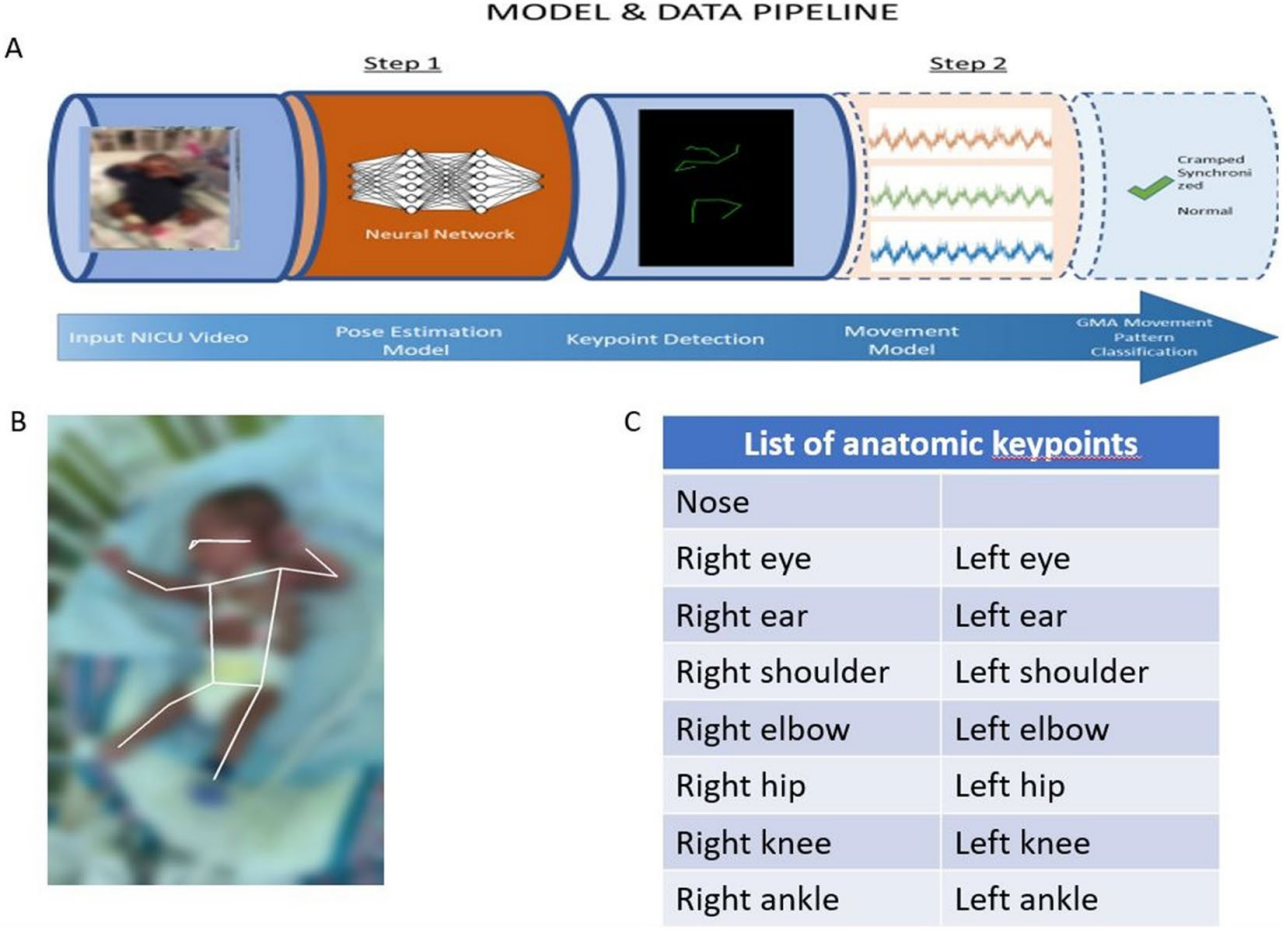
Data pipeline using a two-step framework. (**A**) Model and data pipeline illustrating the two-step framework composed of two distinct models. Step 1 consists of a pose estimation model—which is a neural network trained to detect and localize anatomical key points on NICU specific data. Step 2 consists of a preliminary movement model trained to classify movement as normal or CS using the time series information generated by the pose estimation model. (**B**) Example of the data output from a representative patient. The key points form the infant’s skeleton from which movement is analyzed. (**C**) List of the 17 anatomic key points inferred by the pose estimation model.

In order to test the generalizability of the pose estimation model, 76 GMA videos were used: 62 for training, and 14 for testing for an optimal 80:20 ratio^31,32^. Splitting the train/test dataset at the infant level can give evidence to the model’s generalizability. Of note, during the model training phase, the model was blinded to the hold-out test setWe then developed a two-step sequential framework (Fig. 1A) comprised of two distinct models: (1) a CNN model for pose estimation of anatomic key points, and (2) a time-series movement model prototype to analyze the output of the pose estimation CNN. The goal of this two-step framework is to accurately assign a high-risk for CP diagnosis based on the presence of CS GMs prior to NICU discharge. In the first step, the pose estimation model estimates the location of the infant’s anatomical key points (Fig. 1B, C) relative to the video frame. Each frame of a video is processed by the model resulting in a frame-ordered time-series location of these key points. To train a preliminary pose estimation model, we used the Detectron2 framework using a R-CNN X101-FPN backbone model containing 101 convolution layers^33^. In order to train a model on a limited number of infant videos, we employed the transfer learning method^34^. Starting from a model pre-trained on a generic computer vision data set comprised of images of people, the MS COCO dataset^30^. we then fine-tuned the neural network weights on infant videos in the NICU setting (see Supplemental Material for the training parameters).

**Figure 2:**
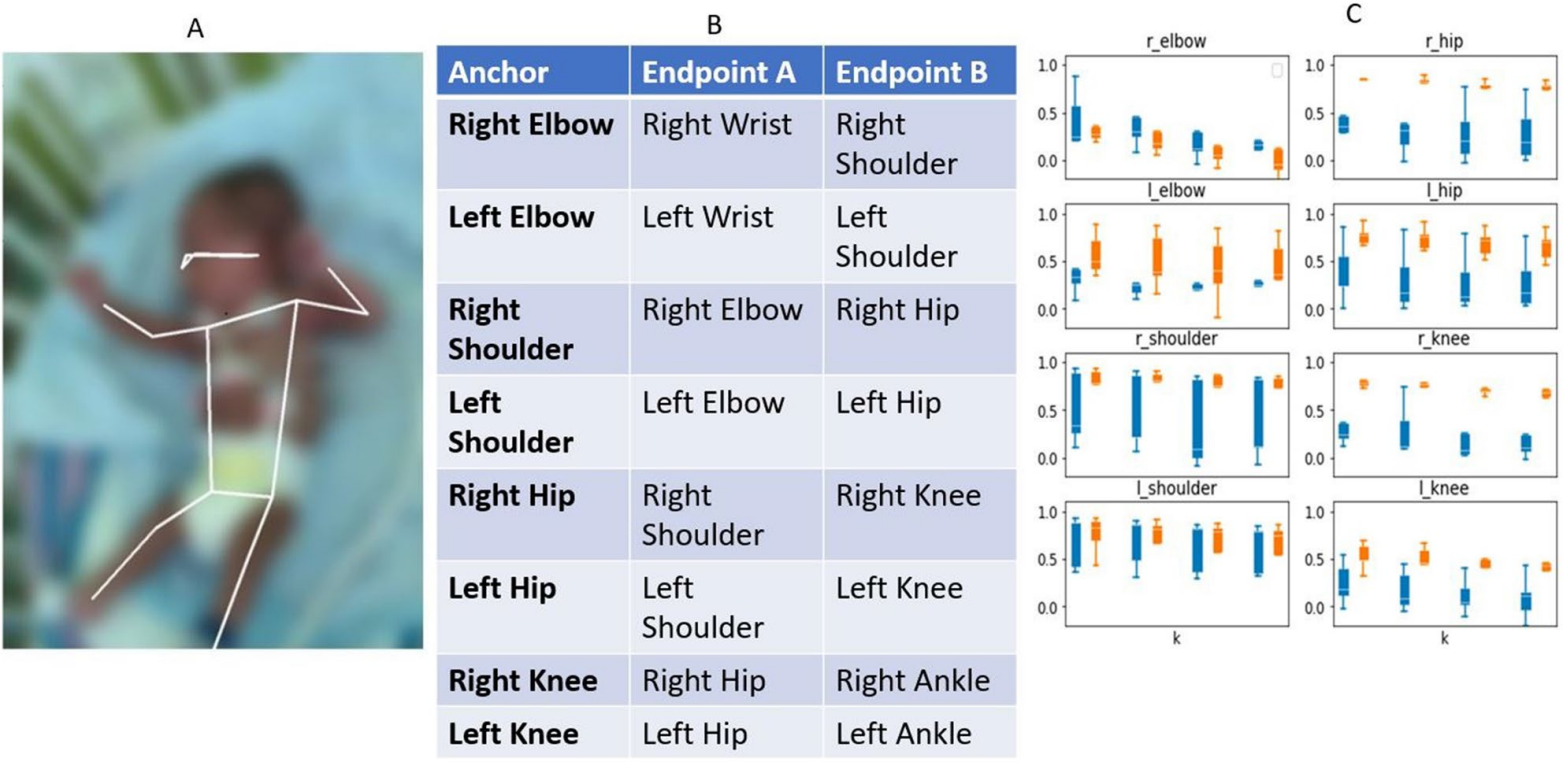
Cosine similarity calculation method. The preliminary movement model (step 2) is trained to measure the cosine similarity between two vectors anchored at the joint. (**A**) Example of a left elbow anchor. The joint angle is calculated using the cosine similarity of the two vectors: (A) left elbow key point to left shoulder key point and (B) left elbow key point to left wrist key point. (**B**) List of the eight joint anchors and their respective endpoints used in the movement model. (**C**) Shows the spread of the autocorrelation values for k = 5, 7, 11, 13 for the eight joints between normal and CS.

In the second step, the preliminary movement model aims to use the time series signals from the pose estimation model to distinguish between normal and CS GMs. Five normal videos (5 videos not included in the test set) and 5 CS videos (1 from the test set and 4 new videos). The movement model converts the inferred poses into eight joint angles (see Fig. 2A) which are then represented using cosine similarity and calculated as follows:

$${d}_{{\text{cosine}}}\left(A,B\right)= \frac{A\cdot B}{\left|\left|A\right|\right|\left|\left|B\right|\right|},$$

where the dot operator ‘$\cdot$’ is the standard Euclidean dot product in two-dimensions and $\Vert \cdot \Vert$ is the Euclidean vector norm. Here, $A$ and $B$ are vectors rooted at the same body joint but pointing at different limb directions. For example, Fig. 2B shows an example of the two vectors used to calculate the cosine similarity value of the left elbow anchor. Note that a cosine similarity value near 1 indicates that the limbs are flexed; whereas a value near −1 indicates that the limbs are extended. A cosine similarity value of 0 means that the limbs are perpendicular (at a 90° angle). Using these time-series cosine similarity representation, we then tracked these angular values across time to visualize patterns within the context of the GMA. Figure 2C shows the spread of the autocorrelation values for K = 5, 7, 11, 13 between the two groups.

These cosine similarity time series were then analyzed using autocorrelation to distinguish movement patterns that indicate high-risk for CP. Autocorrelation represents the degree of similarity between a given time-series and a lagged version of itself over successive time intervals. This study measures the autocorrelation of each angular position for each limb. Autocorrelation measures the relationship between a variable's current value and its past values. When the autocorrelation in a time series is high, it becomes possible to predict future values by referring to past values—indicating GMs that are repeated and predictable. We used a normalized autocorrelation measure to compare across subjects. A normalized autocorrelation of + 1 represents perfect positive correlation, while an autocorrelation of 0 represents very high dissimilarity. The normalized autocorrelation, ${r}_{k}$ was calculated as follows:

$$r(k)=\frac{\sum_{t=k+1}^{T}({y}_{t}-\overline{y })({y}_{t+k}-\overline{y })}{{\sum }_{t=1}^{T}{\left({y}_{t}-\overline{y }\right)}^{2}}$$

where ${y}_{i}$ is autocorrelation at lag $i$, $k$ is the time lag, and $n$ is the number of observations in the time series, and ${y}_{t}$ is the position at time $t$ and  $\overline{y }$ is the average of ${y}_{t}$ for all t.

### Data analysis and metrics

This section describes the evaluation metric used to measure the performance of model using the annotated test set. We measured the localization accuracy of the key point detection model trained on the NICU dataset with the object key point similarity (OKS) of the model’s inference against ground truth ^35^.

OKS was calculated as follows:

$$OKS = exp\left(-\frac{{d}_{i}^{2}}{{s}^{2}{k}_{i}^{2}}\right)$$

where ${d}_{i}$ is the euclidean distance between ground truth and predicted key point location of the ${i}$ th key point; $s$ is scale and ${k}_{i}$ per-key point constant that controls fall off. More specifically, the distance, ${d}_{i},$ is the distance between the pixel location of the model inference and the pixel ground truth evidence provided by human annotators.

The OKS metric shows how close a predicted key point is to the true key point (value from 0 to 1). The greater the value the closer the prediction is to the ground truth. We chose OKS because it is the standard metric to evaluate key points individually^36^.

### Statistical analysis

To statistically measure the difference between the autocorrelation of normal and CS GMs, we performed an independent two sample t-test between the autocorrelation values of normal vs. CS GMs at certain lag level, $k$. For this work, we performed a t-test on autocorrelation values at lag level $k=1, 2, \mathrm{3,5},\mathrm{7,11,13}$ seconds which was empirically sampled as no standard exists in the literature. Results are shown as mean ± SD unless otherwise specified.

### Consent

This study was approved by the University of Virginia Human Sciences Research Internal Review Board (UVA-HSR) with waiver of consent. Patient consent was not required for this study.

## Results

### Cohort demographics

**Figure 3:**
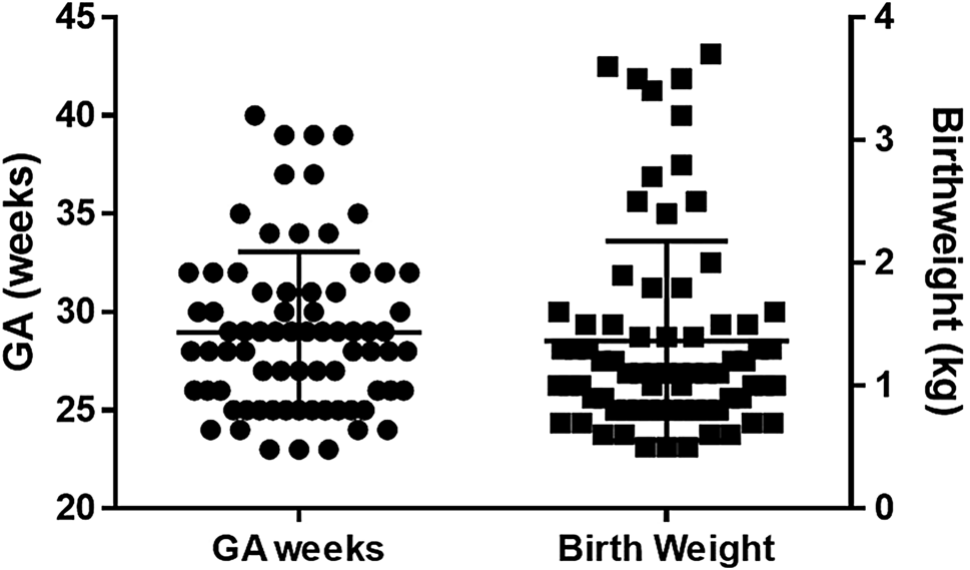
Distribution of gestational age and birth weight of the cohort. The mean GA at birth was 29.9 ± 4.1 weeks (range 23–40 weeks) and the mean birth weight was 1803 ± 387 g.

A total of 85 archival videos from 74 infants were used in this study. Infants’ demographic and clinical information are shown in Table 1. The mean GA at birth was 29.9 ± 4.1 weeks (23–40 weeks); Fig. 3. The analyzed GMA videos were obtained at 35.9 ± 4.6 weeks postmenstrual age (PMA). The majority of the infants (58; 78.4%) were classified as having poor repertoire GMs patterns while 11 (14.9%) and 5 (6.8%) were classified as normal and CS, respectively.

**Table 1:** Cohort demographics and clinical characteristics.

Demographics (n = 74)	
GA (mean ± SD)	28.9 ± 4.1 weeks
Birthweight (mean ± SD)	1803 ± 387 g
Born at less than 32 weeks	57 (77%)
Male gender	46 (62%)
Delivery mode (cesarean section)	45 (61%)
Skin color (White)	50 (68%)
Ethnicity (non-Hispanic)	70 (95%)
PMA at time of first assessment (mean ± SD)	35.9 ± 4.6 weeks
Comorbidities	
sIVH/PVL	9 (14%)
NEC/SIP	5 (8%)
Sepsis	14 (22%)
Severe ROP	10 (14%)

*GA* gestational age, *NEC* necrotizing enterocolitis, *PMA* postmenstrual age, *PVL* periventricular leukomalacia, *SIP* spontaneous intestinal perforation, *sIVH* severe intraventricular hemorrhage-grade 3/4.

To evaluate the pose estimation model, 62 videos (620 frames) were used for developing the anatomical key point detection model and 14 videos (140 frames) served as the test set for measuring key point accuracy. Subsequently, five videos classified by the interprofessional GMA clinical team as normal (none of the normal videos were included in the test set) and five classified as CS GMs (1 video was also included in the test set) were used for the autocorrelation analysis as the basis of an initial preliminary movement model.

### Model performance

**Figure 4:**
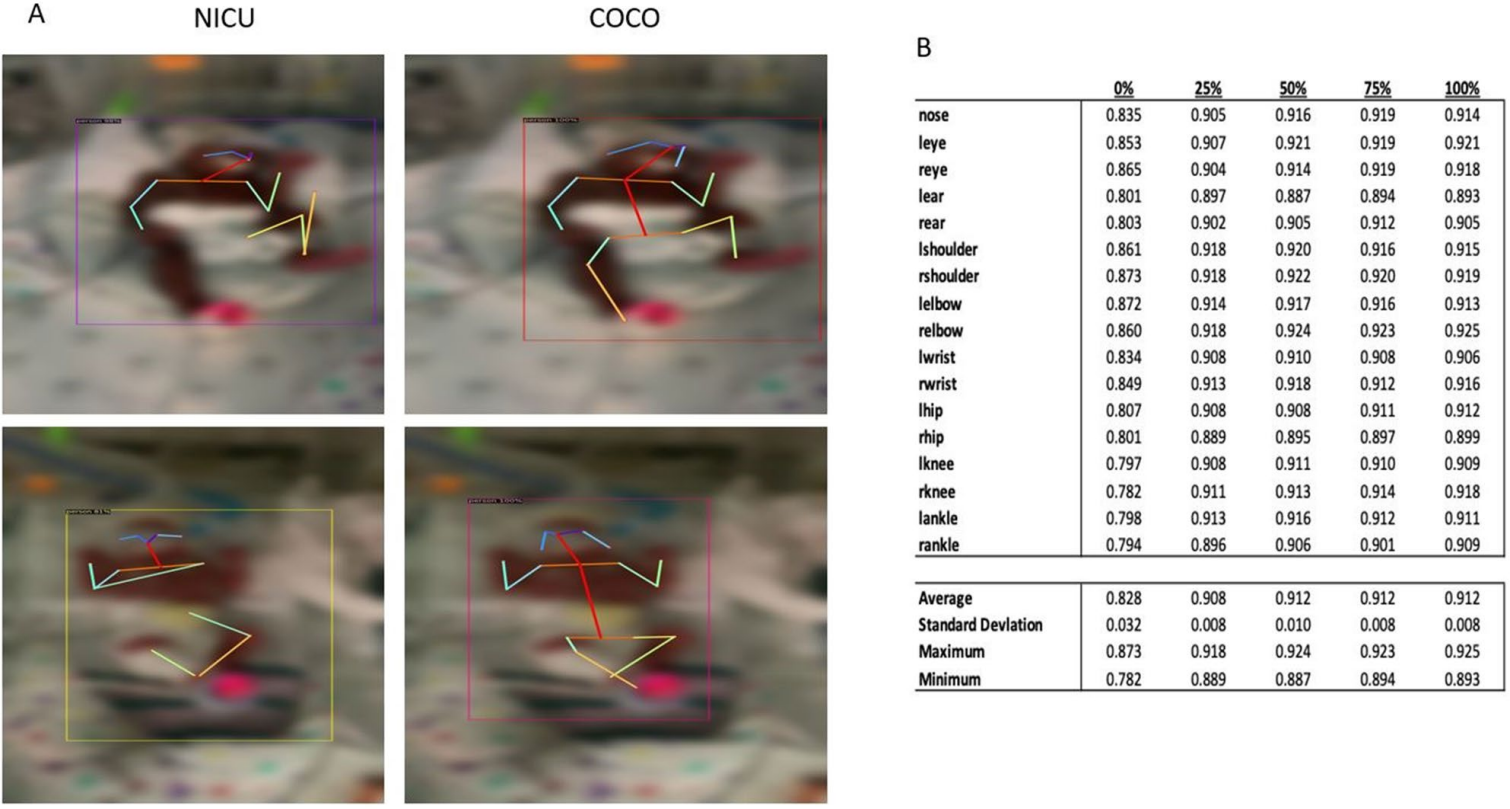
Training of the key point detection model on NICU images. (**A**) Representative example in two patients of the performance of the NICU trained model versus the (MS COCO model) for key point detection. (**B**) OKS results in the NICU-trained versus the MS-COCO models demonstrating a maximum of 0.912 OKS. We performed an ablation analysis where the model was trained on 0% (open-source model), 25%, 50%, 75%, 100% of the available NICU training data and tested each models performance on a hold-out test set. The results show that OKS performance begins to saturate starting at 50% and OKS standard deviation reaches its minimum at 75%. This indicates that additional data would only marginally improve the pose estimation model. For this analysis the following parameters were used: $s=1$ and $k=0.001$ for all 17 anatomic key points. Legend: MS COCO (Microsoft Common Objects in Context); OKS (Object Key point Similarity).

**Figure 5:**
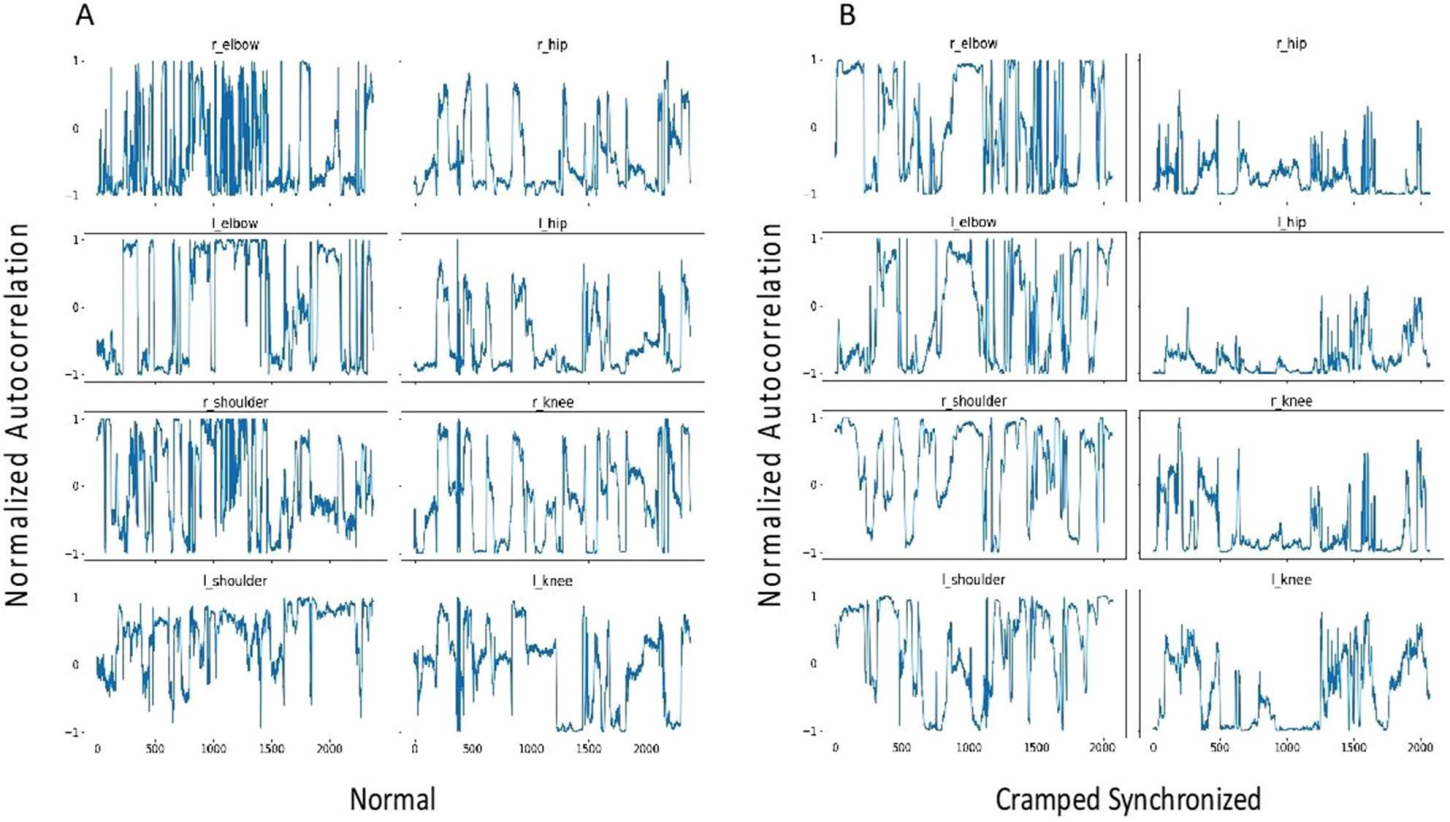
Time series of cosine similarity in normal versus cramped synchronized GMs. Representative examples of time series of cosine similarity for each joint for normal (**A**) and CS GMs (**B**) are shown. The x-axis represents the frame number and the y-axis represents the cosine similarity. A cosine similarity of 0 indicates perpendicular vectors, + 1 indicates vectors with similar orientation and −1 indicates vectors in opposite direction. Because CS GMs lack variability and movements tend to be in extension with activation of limbs at the same time, the signal demonstrates increase in repeated patterns best shown in the left elbow and left knee as well as frequent occurrence of cosine similarity of −1.

**Figure 6:**
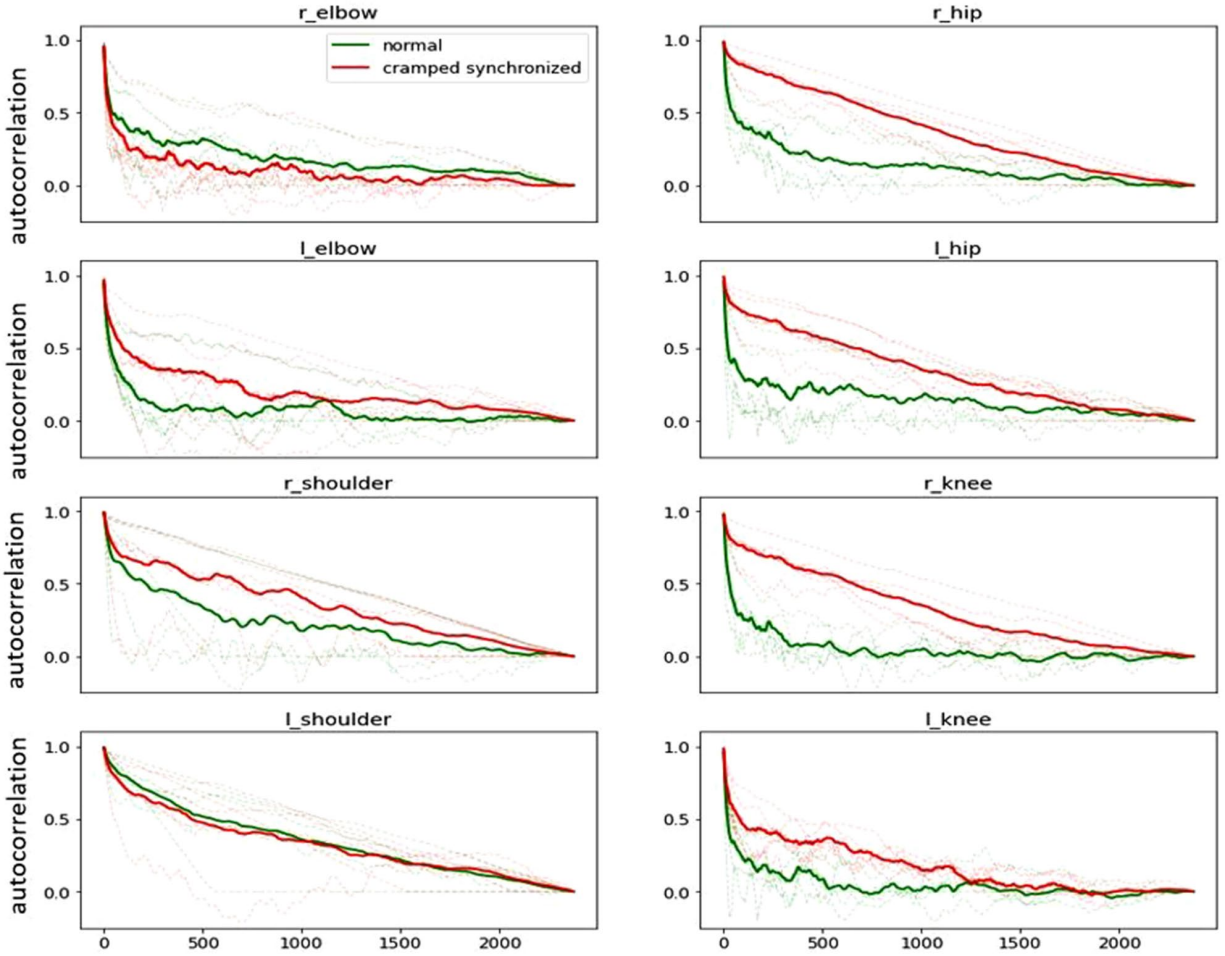
Autocorrelation values in normal versus abnormal GMs. Average autocorrelation values of the eight anatomic anchors for all patients are shown at each lag level (CS GMs are shown in red, normal GMs are shown in green and individual patients are shown in light grey). CS GMs have a higher autocorrelation value at every lag level compared to normal GMs, particularly in the lower extremities.

Our pose estimation model (Step 1) trained on custom NICU data was more accurate (0.91 ± 0.008 OKS) than the MS COCO model (0.83 ± 0.032 OKS, p   ≤ 0.001), representing an improvement in the key point detection accuracy performance of 9% (Fig. 4A). Additionally, improved accuracy was noted for all anatomic key points (Table 2) indicating that the NICU-trained model better adapts to the NICU setting than the MS COCO model. Furthermore, the OKS performance monotonically increased as more patient training data was added (Fig. 4B). The average OKS saturated to achieve a maximum performance of 0.912 beginning at 50% and achieving a minimum OKS standard deviation of 0.008 at 75%. Provided in a supplementary file is a video example of the output of our pose estimation model. The cosine similarity time series of representative infants with normal and CS GMs are shown in Fig. 5. We used the autocorrelation of the time series for each of the eight joints of interest to measure repeated movements. As shown in Fig. 6, the results of the autocorrelation analysis indicate that CS GMs have a higher autocorrelation value than normal GMs in the lower extremity joints. This difference was statistically significant on univariate analysis (Table 3). Of note, a MANOVA test with multiple comparison analysis generated similar results again demonstrating significant differences for the two lag times at 11 and 13 s [p = 0.0418 and p = 0.0357 (data not shown)]. Normal GMs had significantly lower autocorrelation values for lag levels $k= 2, 3, 5, 7, 11, 13$ seconds, which approaches 0 values sharply; whereas, CS GMs had higher autocorrelation values gradually tapering to 0 as lag is increased, indicating a higher measure of “repetitive movement” or movement lacking variety. Normal and CS GMs were maximally distinguished using a lag level of $k= 5, 7, 11$—achieving a p-value less than 0.05 in the lower extremities. There were no statistically significant differences in the upper extremities.

**Table 2:** Differences between COCO model and our NICU model.

	COCO model	NICU model (ours)
Nose	0.83	0.91
Left eye	0.85	0.92
Right eye	0.87	0.92
Left ear	0.80	0.89
Right ear	0.80	0.91
Left shoulder	0.86	0.92
Right shoulder	0.87	0.92
Left elbow	0.87	0.91
Right elbow	0.86	0.92
Left wrist	0.83	0.91
Right wrist	0.85	0.92
Left hip	0.81	0.91
Right hip	0.80	0.90
Left knee	0.80	0.91
Right knee	0.78	0.92
Left ankle	0.80	0.91
Right ankle	0.79	0.91
Mean	0.83	0.91
stdev	0.031	0.008

Our NICU model outperformed and was more accurate than the COCO model representing an improvement in keypoint detection by 9%. p- value < 0.001.

**Table 3:** Autocorrelation values of anatomic key points at varying levels of lag.

Lag in seconds	r_elbow	l_elbow	r_shoulder	l_shoulder	r_hip	l_hip	r_knee	l_knee
1	0.82 (0.44)	−0.98 (0.35)	−1.36 (0.21)	−062 (0.55)	−2.04 (0.08)	−1.89 (0.1)	−1.91 (0.09)	−0.93 (0.38)
2	0.42 (0.68)	−1.65 (0.14)	−1.26 (0.24)	−0.38 (0.71)	−2.36 (0.05)	−1.89 (0.1)	−2.39 (0.04)	−1.23 (0.25)
3	0.5 (0.63)	−1.83 (0.11)	−1.16 (0.28)	−0.29 (0.78)	−2.59 (0.03)	−2.04 (0.08)	−2.83 (0.02)	−1.61 (0.15)
5	0.43 (0.68)	−1.51 (0.17)	−0.91 (0.39)	−0.4 (0.7)	−3.16 (0.01)	−2.49 (0.04)	−3.46 (0.01)	−2.32 (0.05)
7	0.75 (0.47)	−1.3 (0.23)	−0.98 (0.35)	−0.16 (0.88)	−3.61 (0.01)	−2.72 (0.03)	−3.65 (0.01)	−2.34 (0.05)
11	0.49 (0.64)	−0.86 (0.41)	−1.1 (0.3)	−0.0 (1.0)	−3.56 (0.01)	−2.6 (0.03)	−4.2 (0.0)	−2.46 (0.04)
13	0.72 (0.49)	−0.56 (0.59)	−1.02 (0.34)	0.04 (0.97)	−3.6 (0.01)	−2.45 (0.04)	−4.27 (0.0)	−2.21 (0.06)

The independent statistical t-test p-values of autocorrelation results at varying levels of lag (in seconds) for all the anatomic key points are shown demonstrating a statistically significant difference between normal and CS GM autocorrelation values. Statistical significance is indicated by bold text (p < 0.05). Note that a statistically significant difference between normal and CS GMs is only noted in the lower extremities.

*r* right, *l* left.

## Discussion

The GMA is a state-of-the-art tool for early detection of CP. The automated analysis of GMs is of high interest given the many barriers limiting the wide application of the GMA. With this study, we describe a two-step machine learning model to automatically analyze infants’ GMs early, in the writhing stage, expanding previous applications. In the first step, a pose estimation model was able to accurately infer an infant anatomic key point with improved accuracy when compared to out-of-the box pose estimation models. Based on the time series information from the pose estimation model, the movement model prototype was able to accurately separate CS and normal GMs using lower extremity data. We believe that future studies, incorporating features beyond autocorrelation, will improve the model’s ability to accurately classify GMs.

Machine learning techniques are increasingly studied as tools to analyze infants’ movements and previous studies have investigated the utility of computer vision techniques to assess CP risk in infants. Previous studies have evaluated the utility of computer vision techniques to assess CP risk in infants. However, there remains significant gaps in knowledge, specifically with regards to their applicability and utility in the writhing stage of GMs ^16^, as other groups have primarily focused on the fidgety period ^28,37^. Our approach is similar to that of other research groups ^28,37^ and employs a multi-step framework. First infants’ position is tracked and estimated. Then, using the movement information, the risk of CP is determined using a classifier. Our team favored a classical modeling approach in contrast to othertinvestigators who utilized models based on neural network approaches for the classification (prediction) step ^28^. This strategy is more explainable and thereby may provider a higher level of trust and confidence in the results for the end-users. An additional difference in the strategy we employed is the use of custom-trained pose estimation model to improve performance in the NICU environment, characterized by interfering technology (i.e., leads for monitoring, etc.) and clutter. Other studies, also employing classical modeling approaches have used “brittle” models (e.g., optical flow, etc.) to classify infant movements which are less adept for the busy environment of the NICU ^37^.

While studies indicate that abnormal GMs in the writhing stage are less predictive than abnormal GMs in the fidgety stage^6^, determining if earlier identification of risk for CP prior to NICU discharge remains an important goal in order to provide individualized treatment plans upon transition to community resources. Before clinical applications can be considered, further research is needed to better understand differences in movement patterns that can be captured using computer vision and machine learning techniques at different GA and in the writhing phase.

We found that out-of-the-box pose estimation models such as the widely-used MS-COCO dataset, do not translate well to the complex environment of the NICU with sub-optimal performance with regards to anatomic key point localization accuracy (0.83 OKS). The custom trained model we developed using annotated NICU-images demonstrated improved anatomic key points detection accuracy (Table 2). Additionally, we show that increasing the amount of training data thereafter only marginally improved key point localization indicating performance saturation with no further improvement in OKS beyond 75% of the training set.

We also found that the output of the pose estimation model can differentiate between normal and CS GMs using cosine similarity to represent limbs’ angular position and autocorrelation as a measure of repetitive movement. On average, infants with CS GMs had a higher autocorrelation level at every lag factor as opposed to those with normal GMs, indicating increased proportion of repetitive movement patterns. This was only apparent in the lower limbs (hip and knee joints) and maybe due in part to the higher degree of freedom that upper limbs have compared to the lower limbs. To ameliorate this finding, future models will include other measures that may capture high-risk movements, especially those of the upper extremity (i.e. velocity). Finally, the lack of observed difference in the upper extremities could be related to the positioning of the camera and the use of 2D videos obtained as part of clinical care. More advanced video technology may capture additional differences in movement patterns.

There are several limitations to this pilot study. We used a convenience sample of archival GMA videos that were obtained as part of clinical care and not as part of a prospective research protocol. While our institution was an early adopter of the GMA in the NICU and we developed a standardized process for obtaining these videos as part of clinical care, not all videos were optimized and could be used for this study, thus resulting in a limited number of normal and CS videos. Additionally, while poor repertoire is the most frequent GMA pattern in preterm infants, they were intentionally excluded from this phase of the model development ^5^. Future work, incorporating additional vision-based features, will focus on developing a model able to differentiate all writhing GMs, including poor repertroire movements. Our results, based on a single feature (autocorrelation) support the feasibility of such an approach and we plan to develop and test a more robust and complex movement model that includes signals beyond autocorrelation measures and is based on a larger set of videos. Once our data set is expanded, we will be able to optimize the pose estimation model hyper parameters using a validation dataset. Furthermore, we will need to determine the accuracy of this movement model in classifying GMs into three CP risk categories: low (normal GMs), medium (poor repertoire GMs) and high (CS GMs). This classification can then be used to tailor post-discharge follow up recommendations. Finally, validation in videos obtained prospectively, at pre-established gestational ages and varied levels of risk is needed.

## Conclusions

Machine learning techniques are a promising avenue to visualize and objectively analyze infants’ GMs in the writhing stage. A pose-estimation models trained on NICU images can accurately infer infant poses, widening the application of automated GMs video analysis to younger ages. Further, a model built on a single movement feature (autocorrelation) can distinguish CS GMs from normal GMs, supporting further model development in order to classify all writhing GMs patterns. If successful, this approach would provide a one-step visual assessment of GMs and could represent a low-cost method to rapidly screen neonates for CP risk prior to hospital discharge. This is optimal to individualize and optimize follow-up needs as well as therapy recommendations. Importantly, this approach would decrease inequities and allow for infants not born in centers where the GMA is performed routinely to benefit from this assessment. Additionally, this objective tool may be helpful to allow for risk stratification for future interventions based on assigned risk. Future work will focus on improving the current prototype by adding additional vision-based features as well as prospectively validate the model’s accuracy against the state-of-the-art clinical diagnosis.

### Supplementary Information

Supplementary Figure 1.

Supplementary Table 1.

## Data Availability

The dataset collected and/or analyzed for the current study is not publicly available due to patient privacy (videos include infants’ faces), but are available from the corresponding author upon reasonable request.
