# Supplementary material for: Machine learning approaches to evaluate infants’ general movements in the writhing stage—a pilot study
Source: Sci Rep. 2024 Feb 24;14:4522. doi: 10.1038/s41598-024-54297-1 (PMC10894291; doi:10.1038/s41598-024-54297-1)
Supplement: Supplementary file 1 — Supplementary Figure 1. [file 41598_2024_54297_MOESM1_ESM.pptx]

## Slide 1
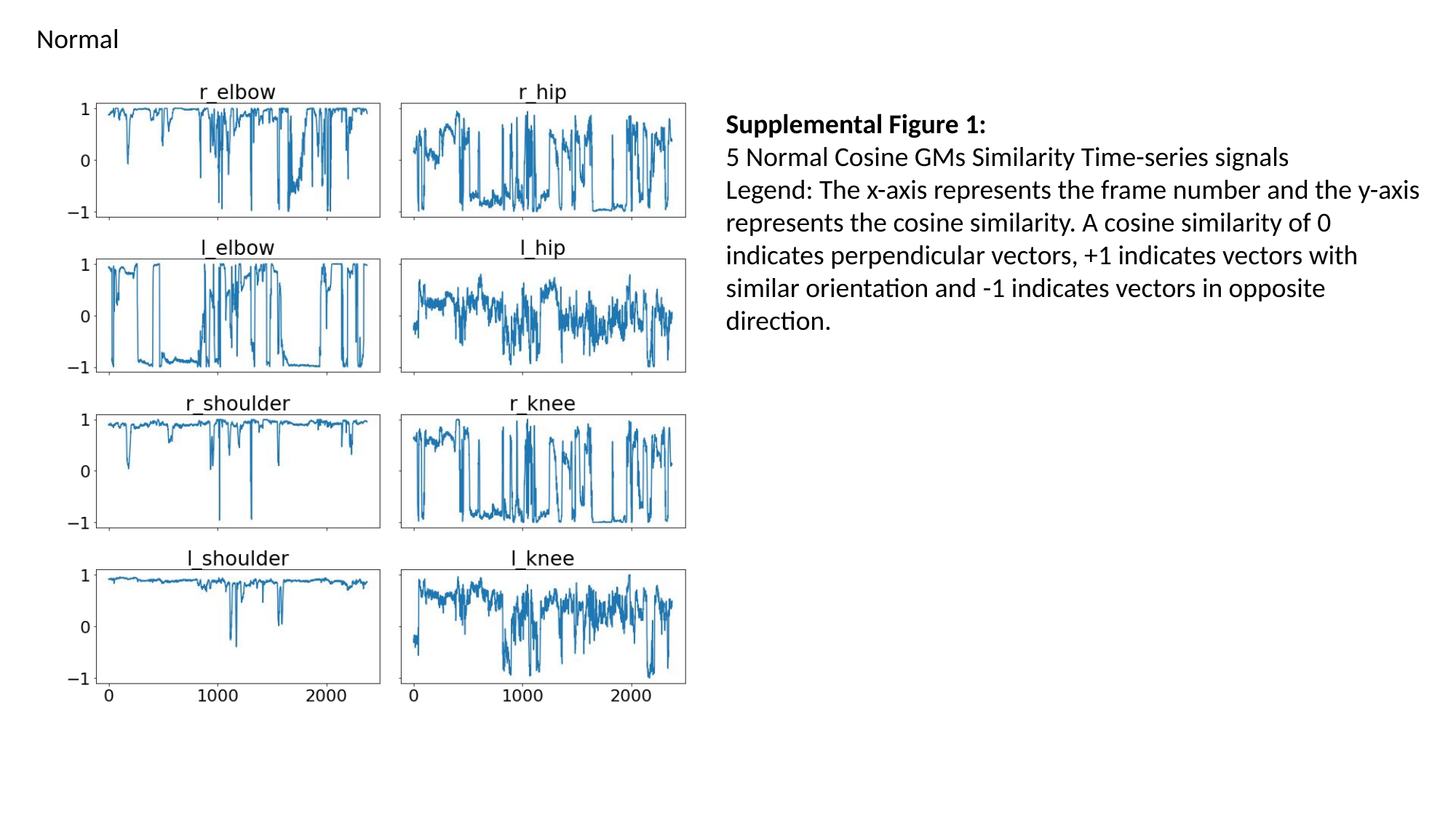

Normal
Supplemental Figure 1:
5 Normal Cosine GMs Similarity Time-series signals
Legend: The x-axis represents the frame number and the y-axis represents the cosine similarity. A cosine similarity of 0 indicates perpendicular vectors, +1 indicates vectors with similar orientation and -1 indicates vectors in opposite direction.

## Slide 2
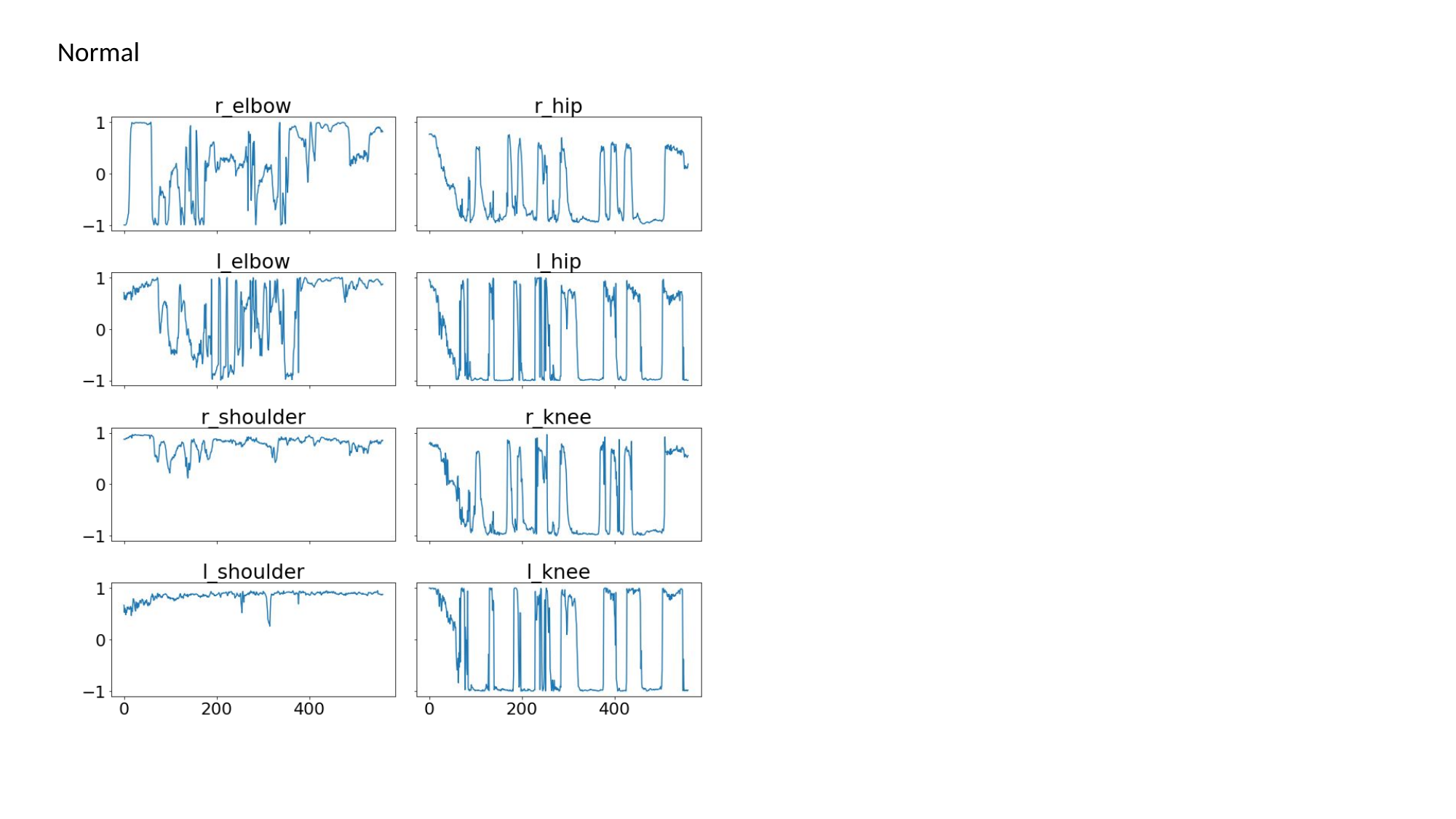

Normal

## Slide 3
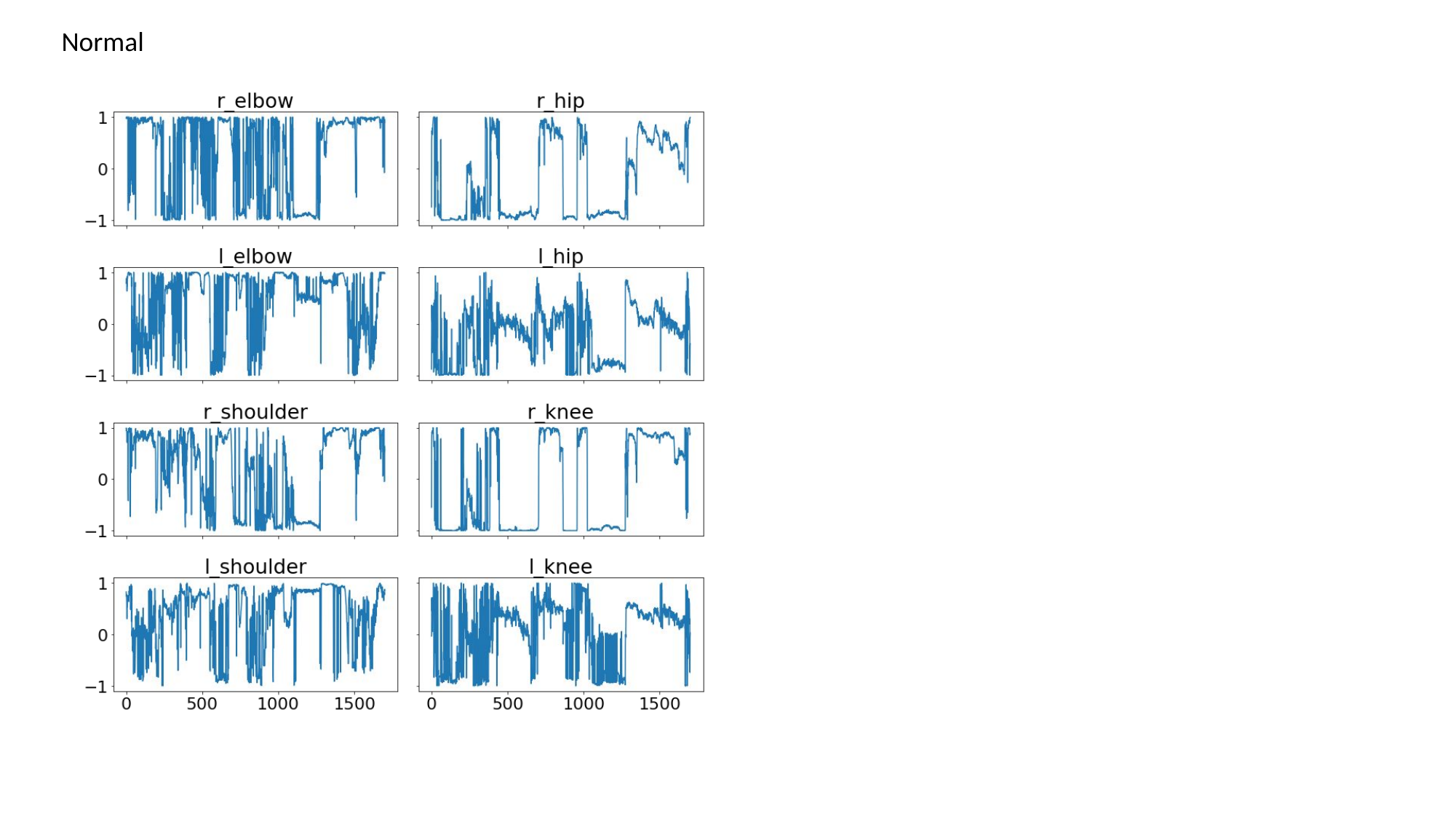

Normal

## Slide 4
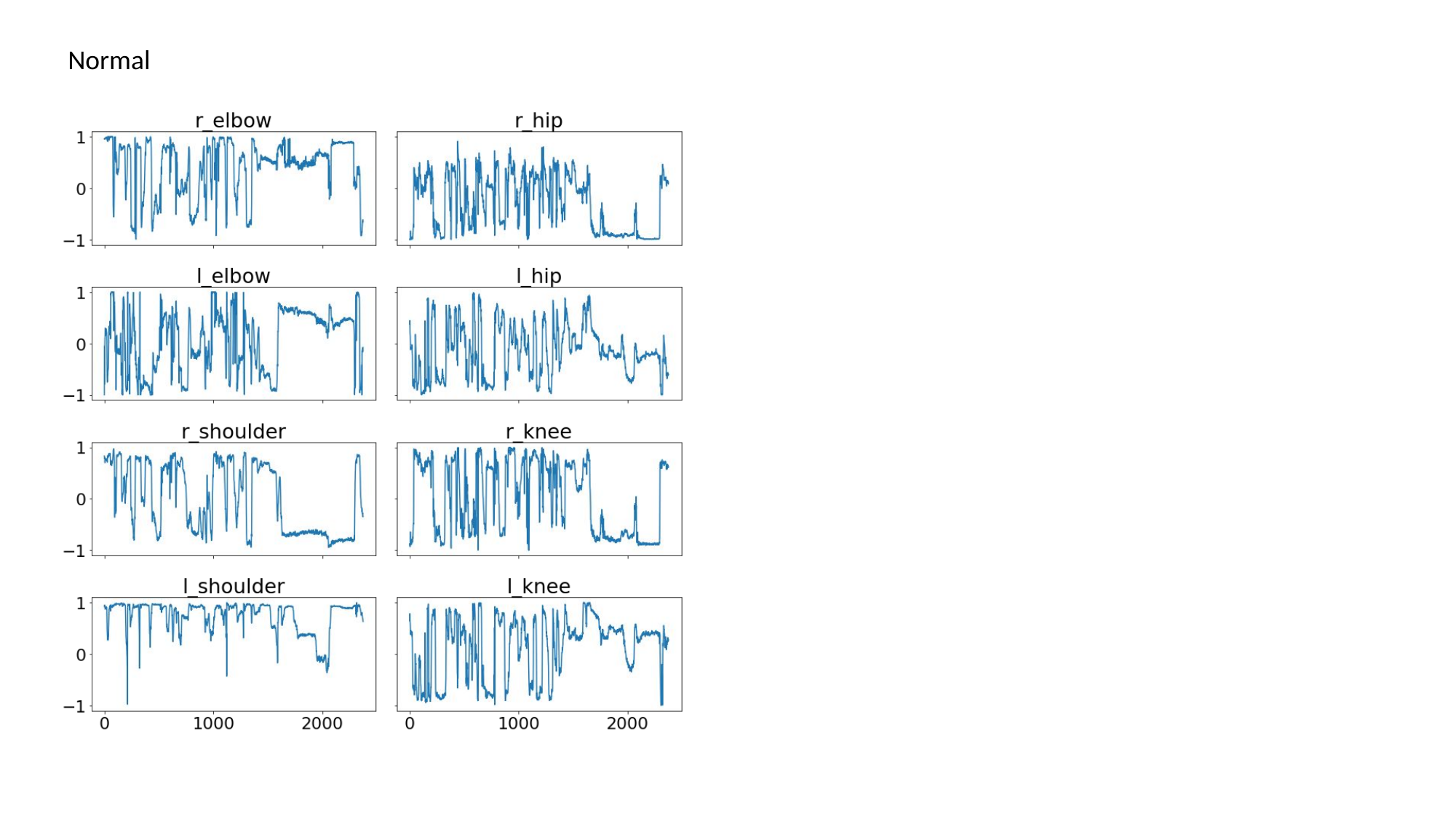

Normal

## Slide 5
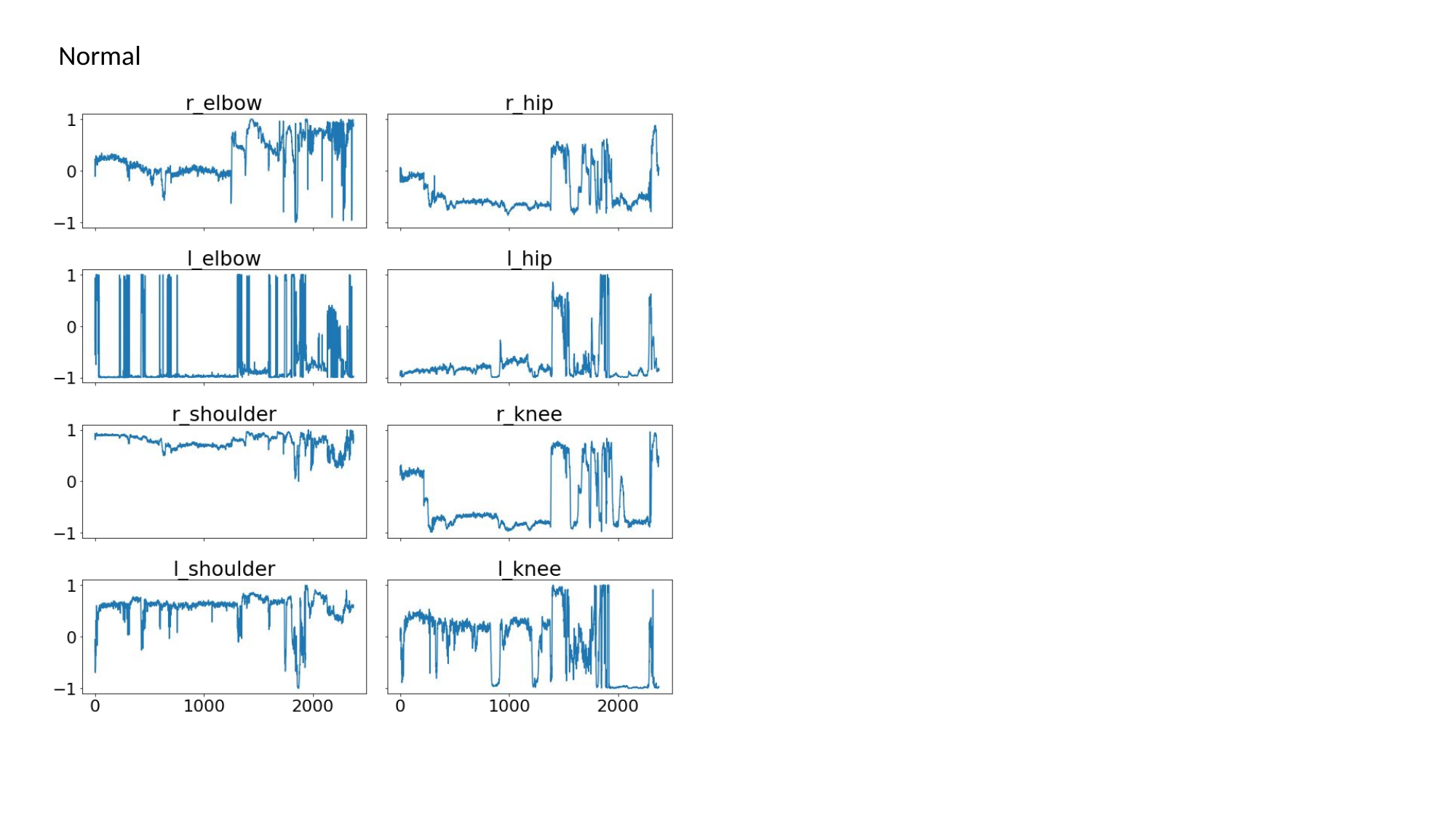

Normal

## Slide 6
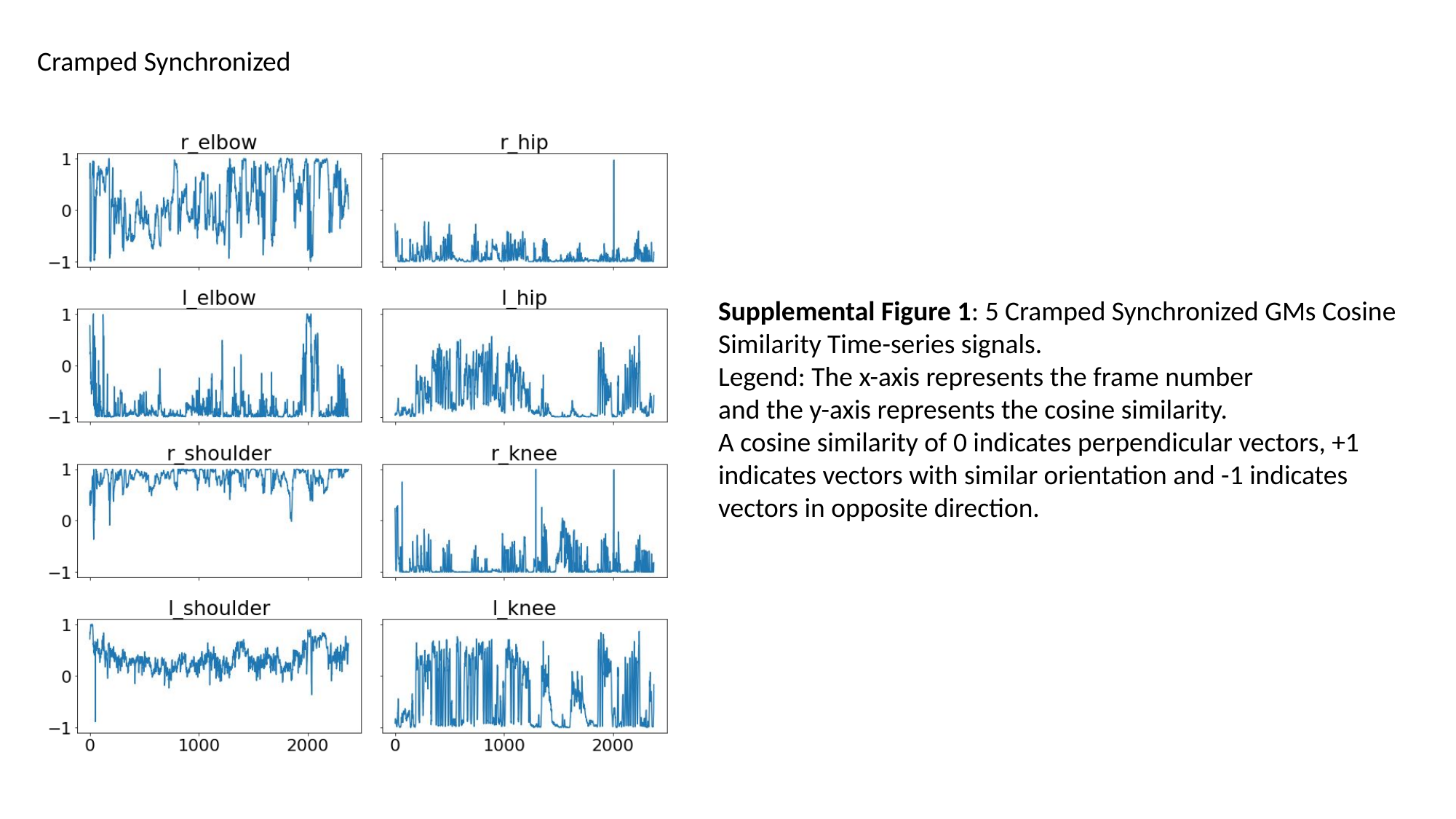

Cramped Synchronized
Supplemental Figure 1: 5 Cramped Synchronized GMs Cosine Similarity Time-series signals.
Legend: The x-axis represents the frame number
and the y-axis represents the cosine similarity.
A cosine similarity of 0 indicates perpendicular vectors, +1 indicates vectors with similar orientation and -1 indicates vectors in opposite direction.

## Slide 7
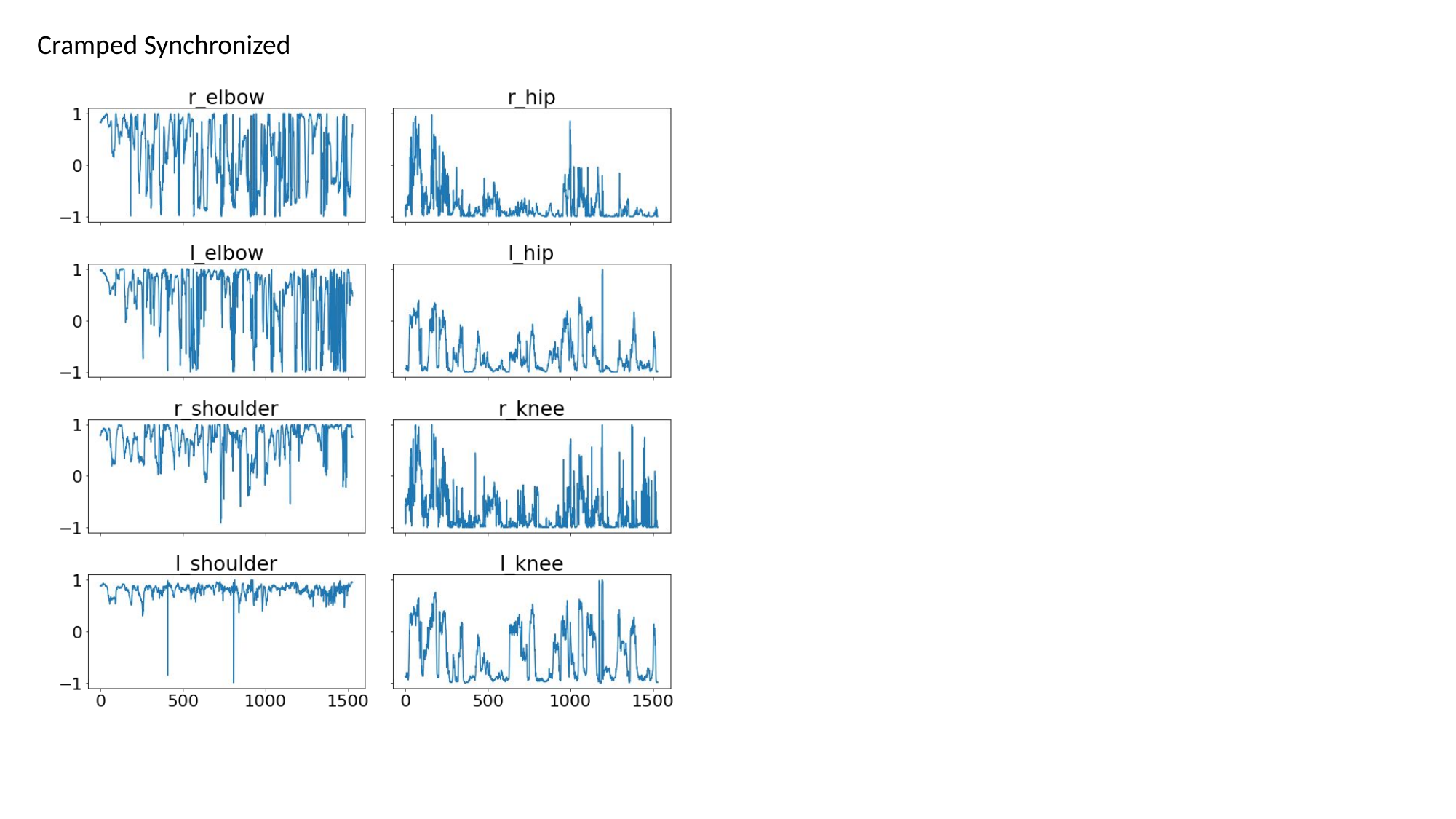

Cramped Synchronized

## Slide 8
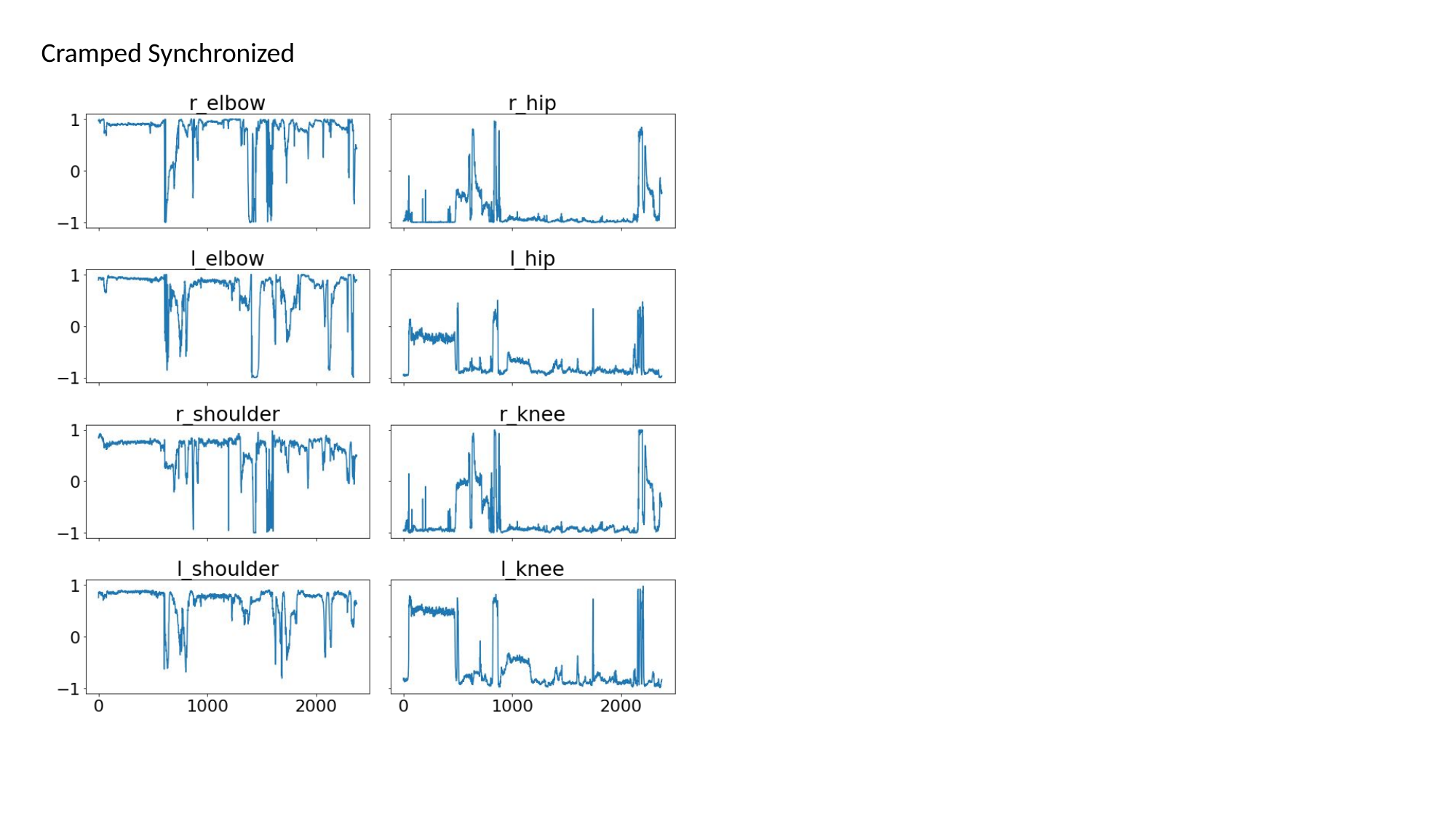

Cramped Synchronized

## Slide 9
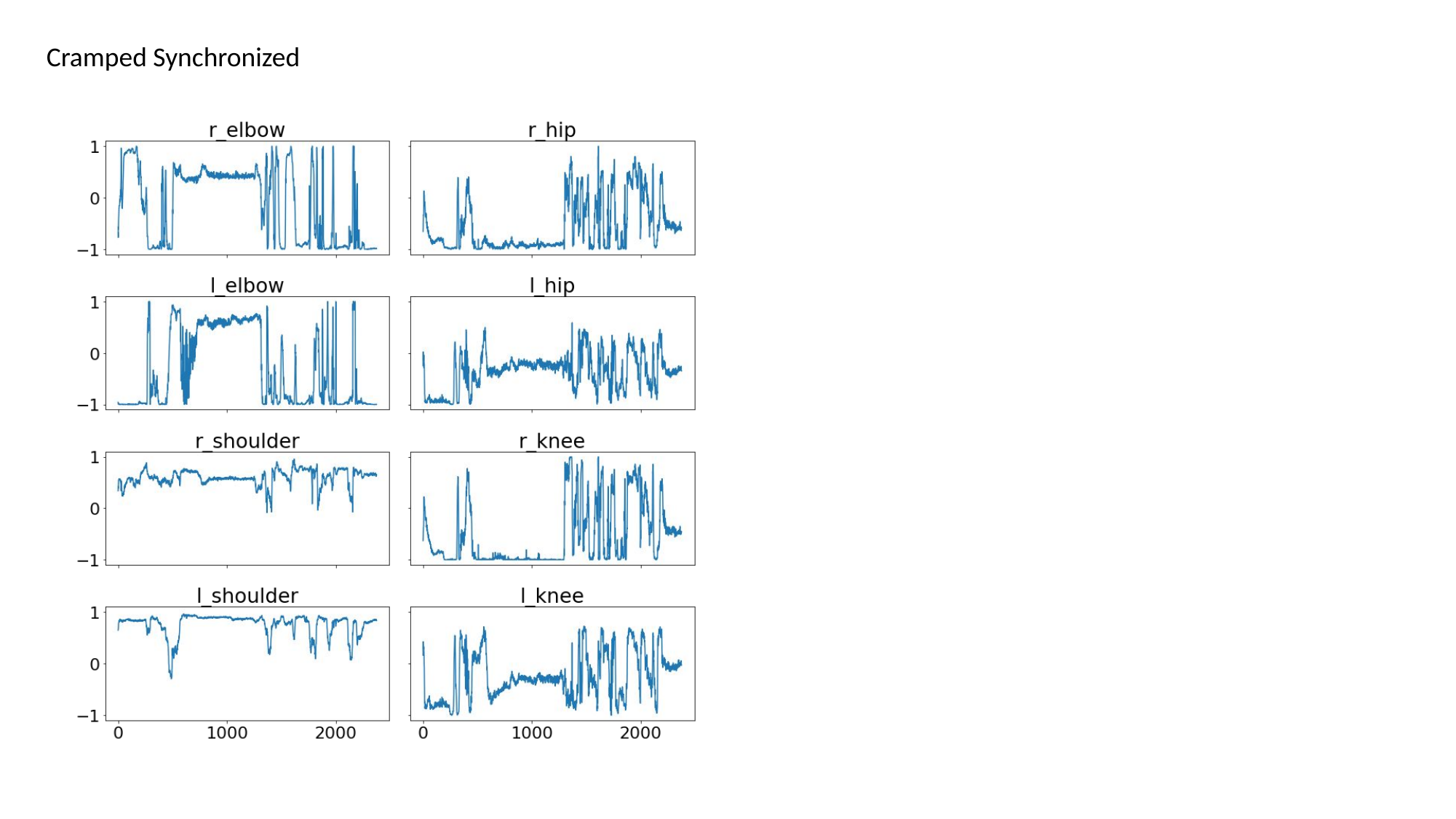

Cramped Synchronized

## Slide 10
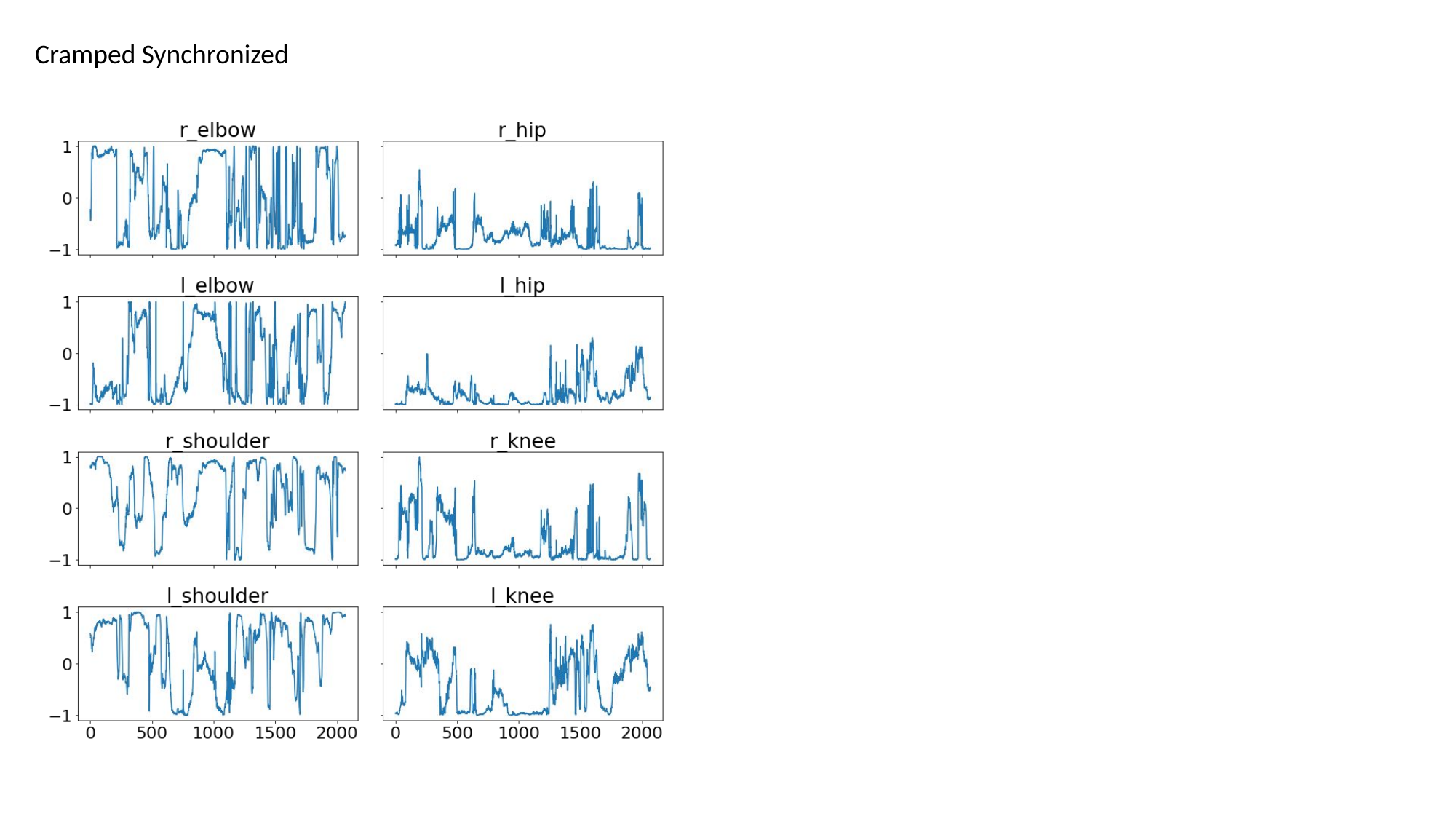

Cramped Synchronized
