# Supplementary material for: Machine learning approaches to evaluate infants’ general movements in the writhing stage—a pilot study
Source: Sci Rep. 2024 Feb 24;14:4522. doi: 10.1038/s41598-024-54297-1 (PMC10894291; doi:10.1038/s41598-024-54297-1)
Supplement: Supplementary file 2 — Supplementary Table 1. [file 41598_2024_54297_MOESM2_ESM.docx]

**Supplemental Table 1: Training Parameters (Detectron2)**
